# ADHD: a critical update for educational professionals

**DOI:** 10.1080/17482631.2017.1298267

**Published:** 2017-05-22

**Authors:** Sanne te Meerman, Laura Batstra, Hans Grietens, Allen Frances

**Affiliations:** ^a^ Department of Special Needs Education and Child Care, University of Groningen, Groningen, the Netherlands; ^b^ Department of Psychiatry, School of Medicine, Duke University, NC, USA

**Keywords:** ADHD, medicalization, behavioural disorders, inclusive education

## Abstract

A medical approach towards behavioural problems could make professionals without a medical background, like teachers and other educational professionals feel inapt. In this article, we raise six scientifically grounded considerations regarding ADHD, currently the most prevalent childhood psychiatric diagnosis. These “need to knows” show just how misguided and potentially stigmatizing current conceptualizations of unruly behaviour have become. Some examples are given of how teachers are misinformed, and alternative ways of reporting about neuropsychological research are suggested. A reinvigorated conceptual understanding of ADHD could help educational institutions to avoid the expensive outsourcing of behavioural problems that could also—and justifiably better—be framed as part of education’s primary mission of professionalized socialization.

## Introduction

Attention deficit hyperactivity disorder (ADHD) is one of the syndromes defined in the Diagnostic and Statistical Manual of Mental Disorders (DSM). In the DSM-5 (American Psychiatric Association, 2013) it is described as a neuro-developmental disorder with a persistent behavioural pattern of severe inattention and/or hyperactivity/impulsivity. The behaviours must be uncharacteristic for the developmental age of the child, be manifest in different settings (for example at home and at school), have started before the age of 12, be present for at least 6 months, and interfere with social and academic performance.

ADHD is currently the most prevalent parent-reported diagnosis among children in the USA (Visser et al., 2014). When DSM-IV was published in 1994 (American Psychiatric Association, 1994) the prevalence of ADHD was an estimated 3% (Goldman, Genel, Bezman, & Slanetz, 1998). Since then, the percentage of children with a parent-reported ADHD diagnosis increased substantially, from 7.8% in 2003 to 9.5% in 2007 to 11.0% in 2011. In 2011, nearly one in five high school boys had been diagnosed with ADHD and about 13.3% of all 11-year-old boys were medicated for ADHD (Visser et al., 2014).

In the USA, the total number of children on ADHD medication skyrocketed from 1.5 million in 1995 (Safer & Zito, 1996) to 3.5 million in 2011 (Visser et al., 2014). Sales of prescription stimulants have quintupled in the last decade (Schwarz, 2013), to well over 11 billion in 2015 (www.jsonline.com, accessed 23 September 2016).

Co-author Allen Frances, who was chair of the DSM-IV, as well as the chair of the DSM-5, David Kupfer, have called the rise in childhood ADHD an “unreal epidemic” (Frances, 2011; Verhoeff, 2010). In an interview in the New York Times (Schwarz, 2013), Keith Conners, a professor emeritus at Duke University who spent much of his career in legitimizing the diagnosis of ADHD, named the rising rates of the ADHD diagnosis in the USA “a national disaster of dangerous proportions”.

Teachers and other school personnel are often the first to suggest the diagnosis of ADHD in a child (Phillips, 2006; Sax & Kautz, 2003). Previous research suggests that teachers tend to feel insecure about dealing with behavioural problems (Walter, Gouze, & Lim, 2006) and hesitant to accept responsibility for students with special needs (Pijl, 2010). In this article, we present six scientifically grounded “need to knows” that unravel misconceptions about ADHD. These topics are selected from a wide array of issues surrounding ADHD because we believe they are the most effective in revealing the catch-all (Singh, 2011) nature of the ADHD classification, and/or the most exemplary of the adverse effects related to the misunderstandings regarding ADHD. We draw mostly from research and practices in the USA, as the epicenter of ADHD (Lloyd, Stead, & Cohen, 2006, p. 3). However, we concur with Richards (2013) that in general “Europe has followed the USA’s lead” by using the DSM-IV and its successor and will also refer to European studies if deemed appropriate. The topics we address are meaningful to teachers and other educational professionals, but certainly also to others such as policy makers who decide on society’s investments in schools.

## 1. Birth month matters

Several studies (Elder, 2010; Evans, Morrill, & Parente, 2010; Halldner et al., 2014; Morrow et al., 2012; Zoëga, Valdimarsdóttir, & Hernández-Díaz, 2012) showed that relative age is a significant determinant of ADHD diagnosis and treatment. Overall, the youngest children in class are twice as likely as their classmates to receive a diagnosis of ADHD and medication. Apparently, health care professionals and teachers tend to classify relative immaturity as ADHD. Additional research has indicated that the majority of general practitioners and teachers are unaware of this association between relative age, ADHD-diagnoses and prescribed medicine (Krabbe, Thoutenhoofd, Conradi, Pijl, & Batstra, 2014). When a child is more restless and less focused than its classmates, teachers should take the child’s relative age into account when judging his/her behaviour. Furthermore, teachers should be aware of the many potential causes of a child’s unruly behaviour. Seeing ADHD as the cause of inattention and hyperactivity is in fact a logical fallacy as it is circular (Erlandsson, Lundin, & Punzi, 2016).

## 2. There is no single cause of ADHD

ADHD is a behavioural description based on criteria that are sensitive to subjectivity and cognitive biases (Gambrill, 2014; Stolzer, 2007). There are no measurable biological markers or objective tests to establish the presence or absence of ADHD (or any other given DSM syndrome). As heuristics, the disorders in the DSM have proven useful in clinical practice and research, especially by creating a common language. Unfortunately, the disorders within these classifications are not generally treated as “heuristic, but (…) have become reified (…) [and] are often treated as if they were natural kinds” (Hyman, 2010). Such “reification” results in circular claims that the behaviour we call ADHD, is caused by ADHD, and that the criteria for “diagnosing” someone are “symptoms” of an underlying mental illness.

Unfortunately, confusing naming and explaining is a common error with regard to behavioural problems (Batstra, Nieweg, & Hadders-Algra, 2014). Seeing ADHD as a brain defect causing problematic behaviour may be tempting: one cause, one solution. However, many factors have been associated with ADHD. These factors may interact and do not always imply causality. They range from divorce (Allen, 2010), poverty (Russell, Ford, Rosenberg, & Kelly, 2013), parenting styles (Johnston, Mash, Miller, & Ninowski, 2012), low maternal education, lone parenthood and reception of social welfare ((Hjern, Weitoft, & Lindblad, 2010), sexual abuse (Weinstein, Staffelbach, & Biaggio, 2000), lack of sleep (Thakkar, 2013), heritability (Larsson, Chang, D’Onofrio, & Lichtenstein, 2013) and perinatal issues (Schmitt & Romanos, 2012) to eczema (Schmitt, Buske-Kirschbaum, & Roessner, 2010), artificial food additives (McCann et al., 2007), mobile phone use (Byun et al., 2013) and growing up in areas with low solar intensity (Arns, van der Heijden, Arnold, & Kenemans, 2013). All these factors and more may play a role when a particular child exhibits impairing hyperactive and inattentive behaviours, and there is no conclusive cause of ADHD.

## 3. Most children with ADHD behaviour have “normal” brains

The classification provided by the DSM, and even the phenomenon of reification can be useful for research purposes (Cromwell, 2010), for example because the categories may facilitate the quest for biological origins of behaviour. And indeed, “case-control” studies -comparing groups of children with and without a diagnosis of ADHD show small group differences in terms of brain anatomy (Sowell et al., 2003) and, in some studies, dopaminergic function (Swanson et al., 2007). However, these differences do not apply to all children diagnosed with ADHD: within-group variation is large, but between-group differences are small and can be demonstrated at group level only (Batstra et al., 2014). In the case of anatomic studies, for example, this means that many with a diagnosis actually have a larger brain than average, while many without a diagnosis have a smaller brain than average. An ADHD diagnosis is a poor predictor of brain size, and brain size is a poor predictor of an ADHD diagnosis.

Furthermore, such individual differences do not refer to a fixed state but to slower anatomical development that mostly catches up later in life (Shaw, Gogtay, & Rapoport, 2010). They are only “abnormal” in the sense that they are less common. They do not reveal any innate defect as is illustrated by the fact that many people with an unusual anatomy or physiology do not experience ADHD related problems.

In addition, one should bear in mind that the groups tested in many brain related studies are rigorously screened and not representative of all those diagnosed with ADHD. These individuals with a so called “refined phenotype” (see also e.g. Horga, Kaur, & Peterson, 2014) are then compared with “supernormal” or “well controls” with no mental disorder and often privileged in other areas of life as well (Schwartz & Susser, 2011; Uher & Rutter, 2012). Although such selection in both patient and control groups might help the search for biological markers, these research findings should not be generalized to children diagnosed in everyday society. The samples do not comprise an accurate representation of their respective populations, meaning an average child with a diagnosis of ADHD and an average “normal” child. This problem is particularly urgent since the DSM 5 has lowered the age of onset criterion, as well as the impairment criterion compared to the previous version, the DSM-IV (Thomas, Mitchell, & Batstra, 2013). Alongside the lowered threshold, the potential to generalize earlier research findings has lowered as well.

The excerpts in Table 1 illustrate that websites with information about ADHD addressed to teachers might not mention the aforementioned limitations of case-control studies. The examples are taken from the top 10 websites for teachers using the search engine Google. Our alternatives, as well as the last example suggest more thoughtful descriptions.

**Table I.** Online information addressed at teachers.

	Findings described as	Source	Preferred message
1	In people with the disorder, these studies show that certain brain areas have less activity and blood flow and that certain brain structures are slightly smaller.	https://www.teachervision.com/learning-disabilities/treatments/30082.html?page = 2	In groups of people diagnosed with ADHD, these studies show that certain brain areas are slightly more likely to show less activity and blood flow and that certain brain structures are, on average, slightly smaller.
42	The researchers found that the brains of boys and girls with ADHD were 3–4% smaller than those of children without ADHD.	http://www.aboutkidshealth.ca/En/ResourceCentres/ADHD/AboutADHD/WhatCausesADHD/Pages/Brain-Differences-in-ADHD.aspx	The researchers found that, on average, the brains of boys and girls with ADHD were 3–4% smaller than those of children without ADHD.
3	Researcher F. Xavier Castellanos found that children with ADHD have subtle brain circuit abnormalities on the right side of the brain in the frontal lobe just behind the forehead.	http://www.educationworld.com/a_issues/issues/issues148.shtml	Researcher F. Xavier Castellanos found that some, but not all, children with ADHD have subtle brain circuit differences on the right side of the brain in the frontal lobe just behind the forehead.
4	As a group, the ADHD children showed 3–4% smaller brain volumes in all regions—the frontal lobes, temporal grey matter, caudate nucleus, and cerebellum.	http://childdevelopmentinfo.com/add-adhd/adhd-causes/	**—**

Note that the research by Castellanos, cited by websites 2–4, uses both refined phenotypes as well as supernormal controls. This means that the average findings are probably not representative for all children with the diagnosis. “ADHD information for teachers” (September, 2016).

## 4. The genetic origins of ADHD may be overestimated

Claims of ADHD heritability are sometimes as high and seemingly accurate as 0.77 (Banerjee, Middleton, & Faraone, 2007). Although activity level might have a substantial genetic basis according to twin studies, “this doesn’t have anything to do with disease” according to Judith Rapoport, researcher at the National Institute of Mental Health (NIMH) (https://www.dnalc.org/view/2198-ADHD-as-a-Genetic-Disorder, accessed 20 December 2016). Furthermore, these heritability claims vary strongly and are subject to debate because of methodological issues of twin, familial and adoption studies that are used for calculating the heritability coefficient. For example, the higher co-occurrence of ADHD between monozygotic twins (who share 100% of their genes) compared to their dizygotic counterparts (who share 50% of their genes) cannot rule out the influence of environment, as homozygote twins are often treated more similarly and more often have a physical and psychological closeness than their heterozygotic counterparts (Furman, 2008). Moreover, these studies still depend on observational tools to assess both parent and child behaviour, and the more sophisticated these tools are (and less prone to rater bias), the lower the estimated genetic effect (Freitag, Rohde, Lempp, & Romanos, 2010). Research into the co-occurrence of ADHD in families suffers from extreme difficulty to separate genetic influences from environmental factors (Furman, 2008) that typically run in families such as poverty, parenting style and divorce (Hjern et al., 2010). Finally, the heritability estimate subsumes the effect of the interplay of genes and environment (Taylor & Sonuga-Barke, 2008).

In genetic association studies that really analyse genetic material and that are more powerful when separating the influence of genetics from other etiologic sources, associated genes show only very small effects (Dillon & Craven, 2014). Combined, they explain less than 10% of variance (Franke, Neale, & Faraone, 2009). This means they occur only slightly more often in diagnosed individuals than in controls, and they do not explain nor predict ADHD behaviours. For educational professionals, this is important to consider as an ADHD label might give a false sense of security with regard to the alleged (genetic) cause of a child’s behaviour and the preferred cure (medication).

## 5. Medication does not benefit most children in the long run

ADHD-related information addressed at teachers, on the internet and in study books, often depicts ADHD as a highly heritable disorder with visible anatomic and neurochemical differences in children diagnosed. (Erlandsson et al., 2016; (Freedman, 2015; Mitchell & Read, 2012). This may have contributed to the rising use of medication over the years. Initially, the widely publicized results of the first MTA (Multimodal Treatment of Attention Deficity Hyperactivity Disorder) study, the largest study in child psychiatry ever, seemed to confirm this biomedical view and the merits of medication. The study suggested that intensive medication management was superior to behavioural therapy as well as combined treatment (MTA cooperative group, 1999). However, follow-up studies of the long-term effects 3 years (Jensen et al., 2007) and 8 years later (Molina et al., 2009) showed that the outcomes between the different experimental groups converged over time, until, on average, no significant difference between medicated and non-medicated children remained after 8 years. Interestingly, the results of the follow-up studies did not nearly draw the amount of attention and publicity as the previous findings did (Nieweg, 2010; Schwarz, 2013). Web of Science indicates that the first results were cited 1483 times while the results after 3 and 8 years were cited 203 and 307 times, respectively. A critical review of the MTA by one of the researchers involved, “Just say yes to drugs alone?” (Pelham, 1999), was cited 56 times only (Web of Science, consulted September, 2016).

Other longitudinal studies also report no long-term benefits (Riddle et al., 2013) or even worse outcomes and adverse effects (Smith, Jongeling, Hartmann, & Russel, 2010) of long-term stimulant use. Hence, while medication may help a small group of children in the long run, most will not benefit from long-term pharmaceutical treatment.

## 6. A diagnosis can be harmful for children

In several countries a confirmed DSM diagnosis opens the door to reimbursement for treatment and school services. This may have promoted “the search for pathology” (Ysseldyke, 2005) in relatively mild cases. US data show that 86% of children diagnosed with ADHD are described as having mild or moderate problems (Visser, Bitsko, Danielson, Perou, & Blumberg, 2010). The question is whether in these mild cases the merits of a confirmed diagnosis—such as acknowledgement of problems and access to help—outweigh possible demerits. Some known disadvantages of a diagnosis are: low teacher and parent expectations that become self-fulfilling prophecies (Pygmalion/Golem effect); prejudice and stigmatization of diagnosed children; children applying stereotypes to themselves, leading to self-stigma and low self-esteem; decline of self-efficacy; a less effective and potentially counter-effective focus on fixed traits instead of behaviours; a more passive role towards problems; difficulties getting life and disability insurances later on in life; and the risk of overlooking contextual, social and societal explanations, due to the specious explanation offered by labelling (Batstra et al., 2012; Cimpian, Arce, Markman, & Dweck, 2007; Heyman & Dweck, 1992; Kamins & Dweck, 1999; Mehta & Farina, 1997; O’Rourke, Haimovitz, Ballweber, Dweck, & Popović, 2014). For these reasons caution is advisable when considering psychiatric classifications for children.

## Conclusion: children need our time, and money

We addressed six issues that educational professionals should be aware of when confronted with inattention and hyperactivity in the classroom. Often, such behaviours are merely the slightly less occurring variations at the poles of any bell-curved behavioural indicator. It is therefore understandable that they are often confused with normal “young” behaviours (paragraph 1). However, disputable yet pervasive claims of ADHD as a genetic neurodevelopmental disorder (paragraphs 3 and 4) could make teachers and other educational professionals feel inapt and might urge them to find solutions outside the realms of their own skills and facilities. Indeed, previous research indicates that teachers are hesitant to accept responsibility for students with special needs (Pijl, 2010). A particular vivid example comes from a teacher in Norway, clearly confusing naming and explaining (see paragraph 2) and expressing hope in the questionable merits of medication (paragraph 5). The teacher finally mentions the eventual marginalization of an unruly child into a separate group, potentially stigmatizing the child (paragraph 6), and effectively defeating the goal of inclusive education:

The diagnosis confirms Roar’s special problems. It’s not me that is wrong or bad or something (…). Now Roar has been given his medicine, and consequently I can expect him to behave properly (…) things are going to be normal again. If not, he’ll be moved to “the group for the badly behaved ones”. (Berg, 2013) cited by Reindal (Reindal, 2016).

For educational professionals, but also for medical and behavioural experts and policy makers, these issues have at least the following implications.

First, more caution is needed with regard to claims made about the etiology of ADHD in general, particularly information addressed at teachers. Generalizing, pathologizing views on the etiology of ADHD-related behaviours—widely dispersed on the internet and in books—do little justice to the different interacting causes of ADHD related behaviours.

Second, we hope that a reinvigorated understanding of ADHD makes us reconsider our own expectations of children. Research indicates that many young children, particularly those diagnosed with ADHD, thrive with more space for physical activity (Song, Lauseng, Lee, Nordstrom, & Katch, 2016), playful learning (Panksepp, 2007), and smaller classrooms (Biddle & Berliner, 2008). Although it might remain necessary to have medical professionals stand by to prevent medical problems being labelled as behavioural in some cases, it is often the other way around. In the absence of proof of ADHD as a clear-cut medical entity, we mostly need to prevent that behavioural problems are unjustly medicalized.

Third, we hope that a reinvigorated conceptual understanding will make teachers and other educational professionals more apprehensive with regard to requesting a diagnosis for a child. Many obtrusive children at risk of falling under the ADHD catch-all umbrella may simply display a difficult temperament. This may have a substantial genetic basis but it is not necessarily a disorder in itself, although it can become one in interaction with an environment that is not sufficiently adapted to the child’s needs. Other children’s unruly or distracted behaviour may be a sign of distress and adverse circumstances. In either case, focusing on these behaviours, and avoiding a disability narrative, is more helpful in teaching these children the behaviours we expect from them. For the tailored approach this entails, we need to provide sufficient (financial) space to the institution and its professionals we entrusted with the larger part of the socialization of our children.
